# Awareness, Knowledge, and Acceptance of Haematopoietic Stem Cell Transplantation for Sickle Cell Anaemia in Nigeria

**DOI:** 10.1155/2016/7062630

**Published:** 2016-09-27

**Authors:** Adewumi Adediran, Modu Baba Kagu, Tamunomieibi Wakama, Aliyu Ahmadu Babadoko, Dapus Obadiah Damulak, Sunday Ocheni, Marcus Inyama Asuquo

**Affiliations:** ^1^Department of Haematology and Blood Transfusion, Faculty of Clinical Sciences, College of Medicine, University of Lagos, Lagos, Nigeria; ^2^Department of Haematology and Blood Transfusion, University of Maiduguri Teaching Hospital, Maiduguri, Borno State, Nigeria; ^3^Department of Haematology and Blood Transfusion, National Hospital, Abuja, Nigeria; ^4^Department of Haematology and Blood Transfusion, Ahmadu Bello University Teaching Hospital, Zaria, Nigeria; ^5^Department of Haematology and Blood Transfusion, Jos University Teaching Hospital, Jos, Nigeria; ^6^Department of Haematology and Immunology, College of Medicine, University of Nigeria, Enugu Campus, Enugu, Nigeria; ^7^Department of Haematology, University of Calabar Teaching Hospital, Calabar, Cross River State, Nigeria

## Abstract

*Background.* Sickle cell anaemia (SCA) is an inherited condition whose clinical manifestations arise from the tendency of haemoglobin to polymerize and deform red blood cells into characteristic sickle shape. Allogeneic bone marrow transplantation offers a cure. The aim of this study was to determine the level of awareness, knowledge, and acceptance of this beneficial procedure in Nigeria.* Materials and Methods.* This multicentre cross-sectional study was conducted in 7 tertiary hospitals in Nigeria in 2015. Approval was obtained from each institution's research and ethics committee. A pretested structured questionnaire was administered to respondents aged 18 years and above and to the parents or guardians of those below 18 years of age.* Results.* There were 265 respondents comprising 120 males and 145 females. One hundred and seventy-one (64.5%) respondents were aware of BMT for the treatment of SCA. About 67.8% (116 of 171) of those who were aware believed SCA can be cured with BMT (*p* = 0.001) and 49.7% (85 of 171) of the respondents accepted BMT (*p* = 0.001).* Conclusion.* Awareness of BMT in Nigeria is low when compared with reports from developed countries. The knowledge is poor and acceptance is low. With adequate information, improved education, and psychological support, more Nigerians will embrace BMT.

## 1. Introduction

Sickle cell anaemia (SCA) is an inherited condition whose clinical manifestations arise from the tendency of the haemoglobin to polymerize and deform red blood cells into characteristic sickle shape [1]. The deformed red cells can block blood flow, leading to pain, organ damage, and stroke.

HbSS is the most common pathological haemoglobin variant worldwide and the majority of children born with SCA die before reaching five years of age [2].

The prevalence of sickle cell trait ranges between 10 and 45% in various parts of sub-Saharan Africa [3]. In Nigeria, carrier prevalence is about 20 to 30% and SCA affects about 2 to 3% of the Nigerian population of more than 160 million [4].

Despite modernization of supportive care, the average life expectancy has remained approximately 40 years for men and women with SCA [5].

Apart from gene therapy which is still in the early phases of testing, successful haematopoietic stem cell transplantation (HSCT) is currently the only established curative treatment modality for SCA [6].

After a successful transplant, disease-free survival (DFS) rates approach 80% to 90% [7].

Though efforts are on to make this procedure safer, some complications associated with it may limit acceptance and eligibility to a selected few.

Eligibility is restricted to patients below 16 years with one or more of the following complications: overt stroke or high transcranial Doppler velocity, debilitating recurrent vasoocclusive episodes, and avascular necrosis or osteonecrosis and cardiopulmonary events such as acute chest syndrome [8].

Due to risks associated with BMT, the decision to recommend the procedure does not simply depend on the patient's diagnosis. A number of variables, including the risk-to-benefit ratio, must therefore be assessed carefully.

Apart from infections due to bacteria, fungi, and viruses resulting from myeloablation and immunosuppression that partly contribute to the major challenges to care-givers, graft-versus-host disease (GVHD) and infertility are additional complications that limit acceptance.

Another challenge to BMT is lack of compatible donors. For over two decades, BMT for SCA has relied on human leukocyte antigen (HLA) matched sibling donors (MSD). Though this option has the best outcome for SCA, it is available to less than 14% of patients with SCA [9]. Cord blood transplantation (CBT) is a suitable alternative, because of less stringent HLA-matching requirements; its use is limited because of low stem cell dose obtainable from a donor and high rate of graft rejection [10].

Another useful alternative is haploidentical transplantation. Today, patients who lack matched related or unrelated donors can now have successful transplantation using this method [11].

Other reasons that limit acceptance of BMT in a third-world country like Nigeria where it is not yet a routine practice include poor understanding of the procedure.

The level of awareness, knowledge, and disposition of patients and their relations or guardians to BMT in Nigeria is not known. This study sought to determine these.

## 2. Materials and Methods

**Figure 1:**
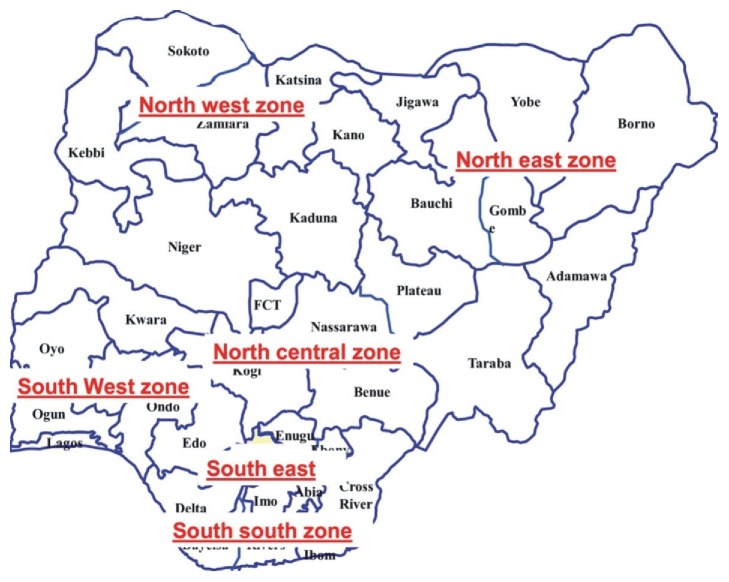
Map of Nigeria showing the six geopolitical zones.

This multicentre cross-sectional study amongst sickle cell anaemia subjects, their parents, siblings, and guardians was conducted amongst tertiary hospitals in Abuja, Lagos, Maiduguri, Enugu, Zaria, Jos, and Calabar in Nigeria. These centres represent Nigeria's six geopolitical zones (North-East, North-West, North-Central, South-West, South-East, and South-South) and the Federal Capital Territory (Figure 1).

**Figure 2:**
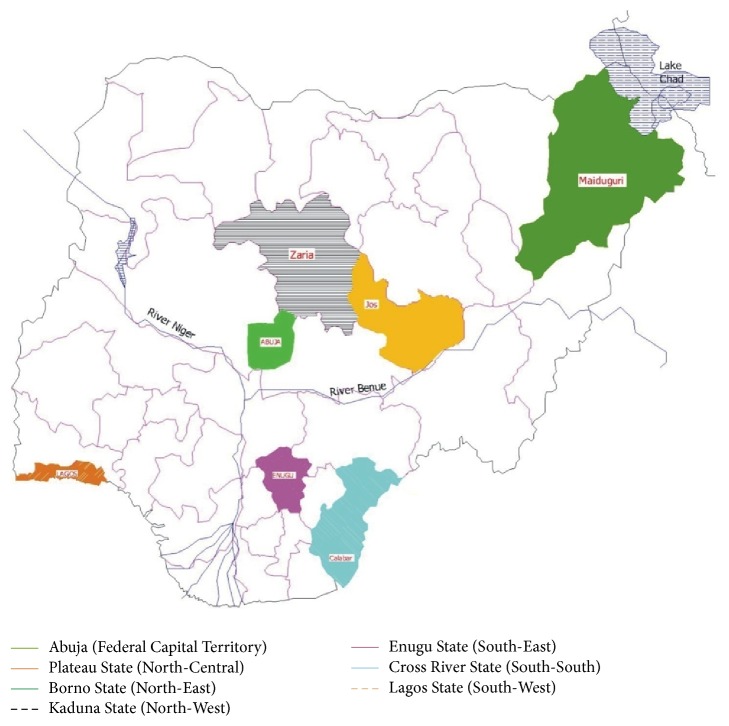
Map of Nigeria showing the states in which the study centres are located.

Abuja is the capital city of Nigeria, in the Federal Capital Territory (Figure 2). It is Nigeria's seat of power where many people of middle and high income classes are found. There are several media houses in Abuja, many of them regularly offering public enlightenment programmes.

Lagos in Lagos State (Figure 2) is located in the South-West geopolitical zone of Nigeria. Until December 1992, Lagos was the capital city of Nigeria. It still remains the economic nerve centre of the country with the largest concentration of industries, financial institutions, and major sea ports. Lagos boasts of several educational institutions and media centres.

Maiduguri is the capital and the largest city in Borno State (Figure 2) in North-Eastern Nigeria. Maiduguri is endowed with many academic institutions and hospitals.

Enugu is the capital city of Enugu State (Figure 2), one of the states in the South-Eastern part of Nigeria. Enugu state is predominantly a civil service state with many traders. Several academic institutions exist in the city with the most popular being the University of Nigeria.

Zaria is in Kaduna State (Figure 2) in the North-Central Nigeria. Zaria's economy is primarily based on agriculture but also has civil servants due to the presence of numerous educational institutions such as Nigerian College of Aviation Technology (NCAT) and Nigerian Institute of Transport Technology (NITT).

Calabar is the capital city of Cross River State (Figure 2) in South-South Nigeria. The city has universities and other educational institutions.

Jos is a city in Plateau State (Figure 2) in the North-Central part of Nigeria. It is the administrative capital of Plateau State. Jos is an important commercial and tourist centre. It is one of the most cosmopolitan cities in Nigeria.

Consenting respondents were recruited at each centre into the study after obtaining each institution's ethics and research committee approval. A three-part, options based questionnaire pretested for congruency and exclusion of ambiguities was administered to 265 (120 males and 145 females) respondents aged 18 years and above and to the parents or guardians of those below 18 years by trained research assistants. The first part tried to assess the level of awareness of respondents about BMT and its use in curing SCA. The second part explored their knowledge of BMT. Some of the questions asked in this section include the following: “Do you believe SCA can be cured?” and “What do you know about BMT?” The third part surveyed the acceptance of the procedure in Nigeria. The level of awareness, knowledge, and acceptance were further assessed for all respondents in each centre and for only respondents who have SCA.

To assess the knowledge, all participants were first scored together according to their response to each question to know how many of them answered each question correctly. Secondly, each participant was scored depending on the number of questions answered correctly. A pass was awarded to a respondent who answered correctly at least five of ten important questions asked. Respondents who were not aware of the existence of BMT were not assessed for knowledge. We also compared the knowledge of the SCA respondents who were aware of the procedure as a cure for SCA and accepted to have the procedure with that of those who rejected the procedure.

### 2.1. Statistical Analysis

Data was analysed using SPSS version 20.0 (Statistical Package for Social Sciences (SPSS), Inc., Chicago, IL, USA). The descriptive data was given as mean ± standard deviation (SD). The Pearson chi-squared test was used to test for association between discrete variables. A *p* value was considered to be statistically significant when <0.05.

## 3. Results

### 3.1. Sociodemographic Characteristics of the Respondents

As shown in Table 1, the age group with the highest frequency was 20–29 years (42.3%), followed by 30–39 years (24.5%). The age group of 50 years and above had the lowest frequency (6%).

After stratification by educational status, respondents with postsecondary education had the highest frequency (52.5%). This was followed by those with secondary education (29.8%) while those who had no education had the lowest frequency (8.3%).

Of the 265 respondents, 178 (67.2%) were single and 83 (31.3%) were married while 4 (1.5%) were widows. Only 4 (1.5%) and 12 (4.5%) of the respondents smoked cigarettes and drank alcohol, respectively.

### 3.2. Awareness of BMT

The association between respondents with or without awareness of BMT and sociodemographic characteristics is also shown in Table 1. One hundred and seventy-one (64.5%) consisting of 72 males and 99 females were aware of the existence of BMT for the treatment of SCA. Although, in all the age groups, those who were aware were more, the age group with the greatest awareness was the age group of 50 years and above where 14 out of 16 (87.5%) were aware of the existence of BMT. A similar trend was seen in educational groups and marital status where those who were aware were more with a single exception in the secondary school category where the reverse was noticed.

Fifty-two percent (89 of 171) of the respondents were made aware of BMT by health workers and 4.1% (7 of 171) were informed by sickle cell patients while 43.9% (75 of 171) by other means.

### 3.3. Assessment of Knowledge of Respondents of BMT

The assessment of knowledge of respondents who were aware of BMT according to their response to each question is shown in Table 2. A significant number of respondents answered correctly that BMT could cure SCA. Of 171 respondents, 67.8% (116 of 171) were correct in their response to the question. Furthermore, about 62% (106 of 171) of them answered correctly to the question “who is the best person to donate?” while 53.8% (92 of 171) also answered correctly to the question on the benefits of donation. However, their knowledge of the procedure was very poor as observed in responses to the following questions, with percentage of correct answers shown in brackets: “What do you know about BMT?” (19.3%); “Does everyone who receives BMT have complication?” (39.8%); “Which of the complications do you fear most?” (3.5%); “What is the major challenge to BMT?” (13.5%); and “What is the estimated cost of BMT?” (19.3%).

### 3.4. Acceptance of BMT

The test of association between all participants with or without awareness on bone marrow transplantation (BMT) and acceptance of BMT is shown in Table 3. About 49.7% (85 of 171) of respondents would want to or have their children or wards undergo BMT. *p* = 0.001. A percentage of 31.6% (54 of 171) could not make up their minds, 15.2% (26 of 171) rejected the procedure, and 3.5% (6 of 171) gave no response.

### 3.5. Awareness, General Knowledge, and Acceptance of BMT for Each Centre

Shown in Table  4 (in Supplementary Material available online at http://dx.doi.org/10.1155/2016/7062630) is the breakdown of the level of awareness, general knowledge, and acceptance of BMT for each centre. Respondents from Abuja with awareness of 86% (43 of 50) and Jos with awareness of 38.5% (15 of 39) had the highest and lowest levels of awareness, respectively. Abuja, the centre with the highest level of awareness, was followed closely by Calabar with awareness of 84.4% (27 of 32).

The centre with the highest level of knowledge was Calabar 81.5% (22 of 27) while Zaria 17.9% (5 of 28) scored the lowest.

The acceptance was highest in Enugu where 9 of 12 (75.0%) agreed to the use of BMT in the treatment of SCA. This was followed by Jos with 66.7% (10 of 15). The centres with the least acceptance were Maiduguri and Zaria that had 34.5% and 35.8%, respectively.

### 3.6. Sickle Cell Anaemia Respondents' Level of Awareness, General Knowledge, and Acceptance of BMT

The breakdown of SCA respondents from each centre is shown in Table  5 (Supplementary). One hundred and eighty-one (68.3%) SCA respondents took part in this study. The centre with the highest percentage of SCA respondents was Enugu where all the 23 respondents had SCA. This was followed closely by Jos with 94.9% (37 of 39). The centre with the lowest was Calabar with 25% (8 of 32).

Of the 181 SCA respondents, a total 106 (58.6%) respondents were aware of BMT at the time of this study. The centre with the highest level of awareness amongst SCA respondents was Abuja with 81.8% (27 of 33) followed by Calabar with 75% (6 of 8) while the centre with the least awareness was Jos with 40.5% (15 of 39).

Of those SCA respondents who were aware of BMT, only two centres, Abuja with 51.9% (14 of 27) and Enugu with 50% (6 of 12), had a fairly good knowledge of BMT. The centre with lowest level of knowledge was Lagos with 28.6% (2 of 7).

Acceptance of BMT amongst SCA respondents was highest at Enugu with 66.7% (8 of 12) and lowest at Maiduguri with 23.1% (3 of 13). The percentage of those who accepted BMT with a fairly good knowledge of the procedure was highest at Lagos centre with 75% (3 of 4) and lowest at Maiduguri with 33.3% (1 of 3).

## 4. Discussion

In recent times, the results of HSCT after myeloablative conditioning in children have been very encouraging, with disease-free survival in most studies of approximately 85% and a transplant-related mortality (TRM) rate of <10%. In a report by Lucarelli et al., following a transplant of 11 SCA patients with age range of 2–16 years from HLA-identical, related donors, none of the patients had complications typical of SCA, such as pains, stroke, or acute chest syndrome [12].

With better understanding of the course of SCD in adults, there has been increasing interest in making HSCT a viable intervention in adults. Recently, a novel nonmyeloablative conditioning regimen was reported to be successful in patients with age range of 16–45 years [13].

In Nigeria, access remains part of major limitation to the broader use of stem cell transplantation (SCT) for treatment of severely affected patients with SCA, due to lack of private and government-funded insurance coverage [14].

In this study, awareness of the use of BMT to cure SCD was generally high, with 60% and 68% in males and females, respectively, and general awareness of 64.5%. This is not surprising because this study was conducted in tertiary health centres in Nigeria. The percentage awareness is however low when compared with reports from developed countries. In a study conducted in 2007 by Chakrabarti and Bareford in the United Kingdom, 93% of their respondents were aware of BMT [15].

In all the age groups, those who were aware were more, and the age group with the greatest awareness amongst the age groups was the age group of 50 years and above where 87.5% were aware of the existence of BMT, similar to the trend seen in educational groups and marital status. This finding could be a reflection of knowledge gained with age through years of visits to medical facilities by this group. We found out that awareness varies from one geopolitical zone to another. This could be as a result of socioeconomic and ethnic differences in different zones that make up Nigeria with a population of over 160 million and over 500 ethnic groups. Overall awareness of a little above sixty percent amongst our respondents for a useful treatment modality of a disorder with economic and social encumbrances as well as progressive irreversible complications calls for more efforts at educating the people about this procedure.

Highest awareness was noticed amongst the SCA respondents in Abuja (81.8%) followed by Calabar (75%), the same centres with almost similar high scores (Abuja 86%; Calabar 84%) of awareness when all respondents were grouped together. This may indicate optimal access to information in these two centres. It is not surprising that Abuja centre had the highest level of awareness and knowledge. It is Nigeria's seat of power where many people of middle and high income classes are found. It also has many hospitals and media houses where information about this procedure is easily obtainable.

It is not surprising that the majority of respondents were informed by health workers since access to information and the drive to look for information are very poor in Nigeria. This scenario is at variance with a report by Chakrabarti and Bareford in which, as said earlier, ninety-three percent were aware of allogeneic BMT, but only 6% of patients had been informed about this procedure by their attending physicians [15].

Knowledge of respondents on BMT was generally poor in this study. Many respondents responded appropriately that SCA can be cured by BMT, but a significant number however did not associate any complications with the procedure. Furthermore, while the majority were right in their response to who should be the best donor, many of them did not know the major challenge to BMT. This finding is worrisome, and it buttresses the need to educate patients and relations on the procedure.

We found that though awareness and knowledge were high amongst parents or guardians, acceptance was low. This shows that other factors including emotional factors may influence acceptance and it agrees with a report that the acceptance of BMT in lieu of cure might vary with the underlying condition or adequacy of psychological support [15] and that parental attitudes should be factored into decisions about whether to offer bone marrow transplantation to children with sickle cell disease [16]. Corroborating this is the fact that, despite poor acceptance, only a few (15.2%) of our respondents outrightly rejected the procedure, while 42.6% could not decide.

Low percentage of acceptance (49.7) obtained in this study is similar to a report by Roth et al. with forty-five percent of parents (54 of 119) accepting the procedure and 35% of adolescents (19 of 55) agreeing to likely undergo the procedure if recommended by their haematologist [17]. It is however at variance with the report by van Besien et al. who found out that substantial proportion of adults with SCA are interested in curative treatment, at the expense of considerable risk [18], and that of Dioguardi et al. [19] who concluded that majority of parents and adolescents are willing to accept the current risks associated with matched sibling HSCT for SCA.

## 5. Conclusion

Awareness of BMT in Nigeria is high but relatively low when compared with reports from developed countries. However, the knowledge of the procedure is poor and acceptance is low. With adequate information, improved education, and psychological support, more Nigerians will embrace this beneficial procedure. Parental attitudes should be factored into decisions to offer bone marrow transplantation to children with sickle cell anaemia.

## Supplementary Material

Supplementary materials consists of Table 4 which describes the awareness, general knowledge and acceptance amongst SCA respondents for each centre, Table 5 which describes the awareness, general knowledge and acceptance for all respondents in each centre and questionnaire on Awareness, Knowledge and Acceptance of Haemopoietic Stem Cell Transplantation (HSCT) or Bone Marrow Transplantation (BMT) for Sickle Cell Anaemia in Nigeria.

## Figures and Tables

**Table 1:** Association between all respondents with or without awareness of bone marrow transplantation (BMT) and sociodemographic characteristics.

Variable	Frequency	Awareness of BMT		*χ* ^2^	*p* value
		Yes	No		
Sex				1.965	0.161
Male	120 (45.3)	72 (60.0)	48 (40.0)		
Female	145 (54.7)	99 (68.3)	46 (31.7)		

Total	265	171 (64.5)	94 (35.5)		

Age				8.067	0.089
<20	46 (17.4)	29 (63.0)	17 (37.0)		
20–29	112 (42.3)	67 (59.8)	45 (40.2)		
30–39	65 (24.5)	40 (61.5)	25 (38.5)		
40–49	26 (9.8)	21 (80.8)	5 (19.2)		
≥50	16 (6.0)	14 (87.5)	2 (12.5)		

Total	265 (100)	171 (64.5)	94 (35.5)		

Education				25.012	0.001
Primary	26 (9.8)	15 (57.7)	11 (42.3)		
Secondary	78 (29.8)	34 (43.6)	44 (56.4)		
Postsecondary	139 (52.5)	107 (77.0)	32 (23.0)		
None	22 (8.3)	15 (68.2)	7 (31.8)		

Total	265 (100)	171 (64.5)	94 (35.5)		

Marital status				4.439	0.109
Single	178 (67.2)	108 (60.7)	70 (39.3)		
Married	83 (31.3)	61 (73.5)	22 (26.5)		
Widow	4 (1.5)	2 (50.0)	2 (50.0)		

Total	265 (100)	171 (64.5)	94 (35.5)		

**Table 2:** Knowledge of respondents who were aware of BMT when asked specific questions.

Variable	Correct	Incorrect	Total
Do you believe SCA can be cured?	116 (67.8%)	55 (32.2%)	171
What do you know about BMT?	33 (19.3%)	138 (80.7%)	171
Does everyone that does BMT have complication?	68 (39.8%)	108 (60.2%)	171
*Which of the complications do you fear most?*			
(1) Donor cells attacking host cells	6 (3.5%)	165 (96.5%)	171
(2) Sterility	6 (3.5%)	165 (96.5%)	171
Who is the best person to donate?	106 (62.0%)	65 (38%)	171
Is there any complication after donation?	78 (45.6%)	93 (54.4%)	171
What is the major challenge to BMT?	23 (13.5%)	148 (86.5%)	171
What is the estimated cost of BMT?	33 (19.3%)	138 (80.7%)	171
What are the benefits of BMT?	92 (53.8%)	79 (46.2%)	171

**Table 3:** Test of association between all participants with awareness of bone marrow transplantation (BMT) and acceptance of BMT.

Age group (years)	Frequency	Percentage	*χ* ^2^	*p* value
Would you want to do BMT or allow your child or ward to do BMT?			25.870	<0.001
Yes	85	49.7		
No	26	15.2		
I don't know	54	31.5		
No response	6	3.5		

## Additional Points

Recommendations: The authors recommend that haematologists in Nigeria should routinely include topics on bone marrow transplantation in their lectures to students, during health weeks and university student union weeks. Parents and patients should also be routinely educated on the role of bone marrow transplantation in the treatment of sickle cell anaemia. The Nigerian Medical Association and its affiliated groups such as Nigerian Society for Haematology and Blood Transfusion (NSHBT) as well as Nigerian Group for Blood and Marrow Transplantation (NGBMT) should be encouraged to offer free public health talks on radio and other media to educate the public on the various treatment modalities for sickle cell anaemia.
