# Supplementary material for: Awareness, Knowledge, and Acceptance of Haematopoietic Stem Cell Transplantation for Sickle Cell Anaemia in Nigeria
Source: Bone Marrow Res. 2016 Sep 27;2016:7062630. doi: 10.1155/2016/7062630 (PMC5059514; doi:10.1155/2016/7062630)
Supplement: Supplementary file 1 — Supplementary materials consists of Table 4 which describes the awareness, general knowledge and acceptance amongst SCA respondents for each centre, Table 5 which describes the awareness, general knowledge and acceptance for all respondents in each centre and questionnaire on Awareness, Knowledge and Acceptance of Haemopoietic Stem Cell Transplantation (HSCT) or Bone Marrow Transplantation (BMT) for Sickle Cell Anaemia in Nigeria. [file 7062630.f1.pdf]

Awareness, Knowledge and Acceptance of Haemopoietic Stem Cell Transplantation for Sickle Cell Anaemia in Nigeria.

SUPPLEMENTARY

Table 4: Awareness, General Knowledge and Acceptance amongst SCA respondents for each Centre.

	Abuja	Calabar	Enugu	Jos	Lagos	Maiduguri	Zaria	Total
All respondents	50	32	23	39	25	48	48	265
SCA respondents(%)	33(66)	8(25)	23(100)	37(94.9)	11(44)	28(58.3)	41(85.4)	181(68.3)
SCA+aw (%)	27(81.8)	6(75)	12(52.2)	15(40.5)	7(63.6)	13(46.4)	26(63.4)	106(58.6)
SCA+aw+kn (%)	14(51.9)	2(33.3)	6(50)	6(40)	2(28.6)	4(30.8)	11(42.3)	45(42.5)
SCA+aw+ac (%)	14(51.9)	3(50)	8(66.7)	9(60)	4 (57.1)	3(23.1)	10(38.5)	51(48.1)
SCA+aw+rj	13(48.1)	3(50)	4(66.7)	6(40)	3(42.9)	10(76.9)	16(61.5)	55(51.9)
SCA+aw+ac+kn(%)	7(50)	2(33.3)	5(62.5)	4(44.4)	3(75)	1(33.3)	5(50)	27(52.9)
SCA+aw+rj+kn(%)	7(53.8)	1(33.3)	3(75)	2(33.3)	1(33.3)	1(10)	7 (43.8)	22(40)
Overall ac(%)	14(42.4)	1(12.5)	12(52.1)	22(59.5)	4(36.4)	4(14.2)	12(29.3)	69(38.1)

KEY: SCA-sickle cell anaemia aw-awareness; kn-good knowledge; ac-acceptance; rj-rejection.

Table 5: Awareness, General Knowledge and Acceptance for each centre

	Abuja	Calabar	Enugu	Jos	Lagos	Maiduguri	Zaria	Total
Respondents	50	32	23	39	25	48	48	265
Awareness	43	27	12	15	17	29	28	171
% Awareness	86.0	84.4	52.2	38.5	68.0	60.4	58.3	64.5
Knowledge	26	22	5	5	10	9	5	82
% Knowledge	60.5	81.5	41.6	33.3	58.8	31.0	17.9	30.9
Acceptance	25	14	12	22	9	13	13	110
% Acceptance	50	43.8	52.2	56.4	44.0	27.0	27.0	41.5

Questionnaire on Awareness, Knowledge and Acceptance of Haemopoietic Stem Cell Transplantation (HSCT) or Bone Marrow Transplantation (BMT) for Sickle Cell Anaemia in Nigeria.

SECTION A. (To be filled by all participants)

PARTICIPANT

A. Sickle cell anaemia patient

B. Parent   mother   father

C. Guardian

D. Sibling

1. Age as at last birthday_________

A. < 18 years

B. 18 – 27 years

C. 28 – 37 years

D. 38 – 47 years

E. > 48 years

2. Sex:________

A. Male

B. Female

3. If female, age of starting menstruation_____________

4. Tribe___________

5. Educational status:

A. Primary_____________

B. Secondary__________

C. Post-secondary _______

6. Occupation:

A. Student_______

B. Unemployed_______

C. Employed (civil servant)________

D. Retired____________

Social History

7. Cigarette smokingA. Yes______ B. No_______

8. Alcohol intake A. Yes_______ B. No_________

Marital Status

A. Single
B. Married
C. Widow

SECTION B. (For participants with Sickle Cell Anaemia patients ONLY)

9. Genotype_________, 10. Blood group_________

11. No of siblings with genotype A. AA_______ B. AS_________C. SS_________

12. How often have you been hospitalised in the last one year?

A. Once_______ B. Twice_______ C. 3 times________ D. More than 3 times_________

13. Number of transfusions per year________

14. Number of crises per year_________

15.Which of the following sickle cell complications do you have in the past?

a.Too low haemoglobin and frequent transfusion b. Severe pain c. Leg ulcer d. Recurrent priapism

e. Kidney problemf. Lung problem g. Stroke h. Gall Bladder disease i. Eye problems j. Joints

Others (please specify) ____________________

SECTION C (To be filled by all participants)

16. Do you believe sickle cell anaemia can be cured? A. Yes___ B.No_______C. Don't know_______

17. Have you heard of bone marrow transplantation (BMT) before? A. Yes_______ No_________

18. If yes, who told you? a) A sickle cell patient (b) A health worker

(d) Others Specify _____________________

19. What do you know about BMT? (tick your option)

a. I don't know

b. Infusion of blood cells after collecting it from a suitable donor.

c. A surgical operation done by cutting the bone of a suitable donor and implanting it on the recipient.

d. A procedure whereby stem cells are harvested from a suitable donor and infused into a recipient

20. Who do you think is the best person to donate bone marrow?

(a)Mother to father (b) Mother to a girl (c). Father to a boy (d) Brother to a boy

(e) Sister to a girl (f) Brother or sister to anybody (g) Anybody compatible can donate

21. What problem do you think the donor will have after donation?

(a) Probable death (b) Low haemoglobin (c) Serious illness (d) Healthy

22.Tick any of the complications of BMT you know.

A. Rejection B. Sterility C. Obesity D. Heart diseaseE. Diabetes

F. Donor cells attacking the host cells G. Death H. None of the above I. Not sure

Others (Please specify)____

23. Of all the complications of BMT, which ones do you fear most?________________________

24.Do you believe that not everybody that do BMT suffer from any of these complications?

A. Yes______ B. No________ C.I don't know________

25. Do you know anybody that has done BMT? A. Yes______ B. No_______

26. If yes, what is the outcome? A. Good________ B. Bad_______ C. I don't know_________

27. If bad, what was the problem?____

28. Do you have any idea about the cost of the procedure in naira?

A. No idea_______ B. Less than I million naira______ C. 1-5million naira_______

D. 6-10 million naira_______ > 5 million ____________

29. Would you want to do BMT or allow your child or ward to do BMT?

Yes_____ No____ Don't know_____

30. If Yes, What in your opinion are the benefits? ____

31. If No, give reasons____

32.What do you think is a major challenge to BMT?

A. Lack of compatible donor___B. Lack of awareness______ C. Cost of the procedure_________

D. Fear of complications_________ E. Fear of probable death__________
